# Electrochemical Organic Synthesis of Electron-Rich Biaryl Scaffolds: An Update

**DOI:** 10.3390/molecules26226968

**Published:** 2021-11-18

**Authors:** Fabrizio Medici, Simonetta Resta, Alessandra Puglisi, Sergio Rossi, Laura Raimondi, Maurizio Benaglia

**Affiliations:** Dipartimento di Chimica, Università degli Studi di Milano, Via Golgi 19, 20133 Milano, Italy; simonetta.resta@unimi.it (S.R.); alessandra.puglsi@unimi.it (A.P.); sergio.rossi@unimi.it (S.R.); lauramaria.raimondi@unimi.it (L.R.)

**Keywords:** biaryls, electrorganic synthesis, aryls coupling, electrodes, radical chemistry

## Abstract

Biaryl scaffolds are widely spread in biologically important natural products, in numerous therapeutic agents, but they are also considered a privileged class of ligands and (organo)catalysts; therefore, the development of efficient alternative methodologies to prepare such compounds is always attracting much attention. The present review discusses the organic electrosynthesis of biaryls starting from phenols, anilines, naphthols, and naphthylamines. The most significant examples of the works reported in the last decade are presented and classified according to the single class of molecules: after the introduction, the first three sections relate to the reactions of phenols, naphthols, and anilines, respectively; the other two sections refer to cross-coupling and miscellaneous reactions.

## 1. Introduction

**Figure 1:**
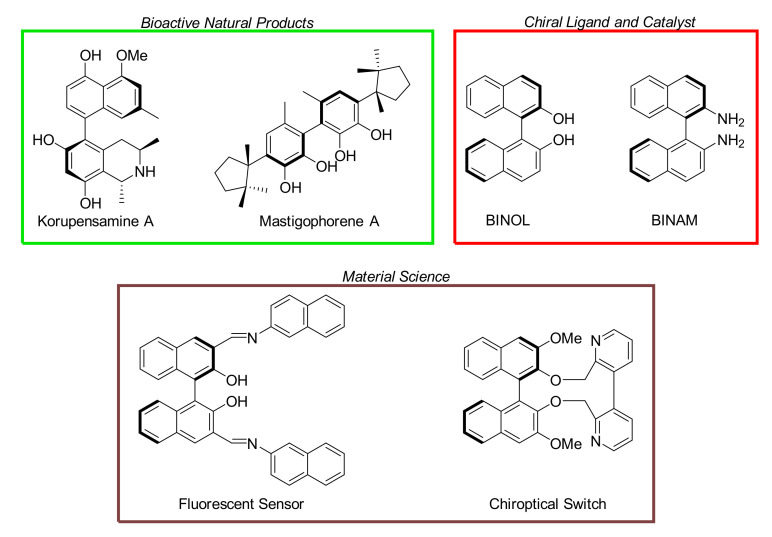
Example of biaryl scaffolds and their applications.

Biaryl systems were demonstrated to be pluripotent scaffolds in different fields. They can be found in a wide range of natural products (Figure 1) [1,2]; they are widely used as ligands in catalysis [3,4], and some of them are important bioactive molecules [5,6] or employed in materials [7] and agrochemicals [8].

Due to the prominent role of the biaryl scaffold, chemists in the last century have put a lot of effort into disclosing different synthetic pathways to assemble it. The possibilities are represented by the Ullmann reaction [9,10], the Gomberg–Bachman arylation [11], transition metal catalysed cross-coupling [12,13], and by the more modern photo-splicing [14].

In the last 30 years, a “new” area of chemistry has started to play a more critical role in the synthesis: organic electrochemistry. As the name suggests, it uses electricity as a driving force to create new bonds and to build molecules in an easy and sometimes greener way compared to traditional methods, opening new and unconventional synthetic avenues out of the reach of the established chemistry [15,16,17]. The use of electrons as actual reagents in the chemical transformation set off the inessentiality of conventional oxidising and reducing agents. Additionally, they are renewable, inexpensive, and safe; fewer reagents implied less waste, meaning a greener reaction [18]. This review focuses on the contributions in the last decade in the area of electrocoupling reactions of phenols, anilines, naphthols, and naphthylamines to afford biaryl systems.

## 2. Biphenols Electrosynthesis

**Scheme 1:**
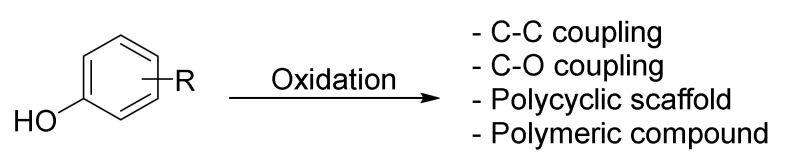
Products mixtures from oxidation of phenols.

Biphenols represent a structural motif crucial in a wide range of natural products [19], ligands [20], and also materials chemistry [21]. The direct oxidation of phenol derivatives, with conventional method or by simple anodic treatment, results in forming a complex mixture of products (Scheme 1).

This problem was overcome with the introduction of the BDD (boron-doped diamond) electrode. It was then possible to generate the hydroxyl radical at a lower potential without generating O_2_ [22,23]. Further help also comes from fluorinated alcohols such as 1,1,1,3,3,3-hexafluoroisopropanol (HFIP); fluorinated media in general, indeed, are able to lower the nucleophilicity of the substrates, such as phenols, due to the solvent shield effect generated through the high number of hydrogen bonding [24].

**Scheme 2:**
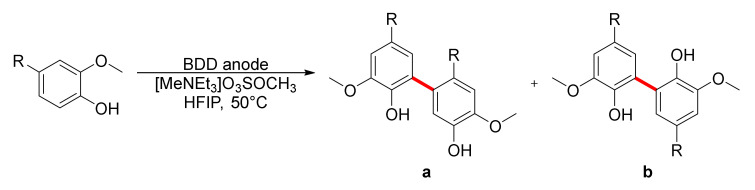
Guaiacol derivatives electrocoupling products: non-symmetrical (**a**), symmetrical (**b**).

One of the first examples that demonstrated the potentiality of the BDD/HFIP couple was published by Kirste et al. (Scheme 2) in the anodic coupling of guaiacol [25].

**Scheme 3:**
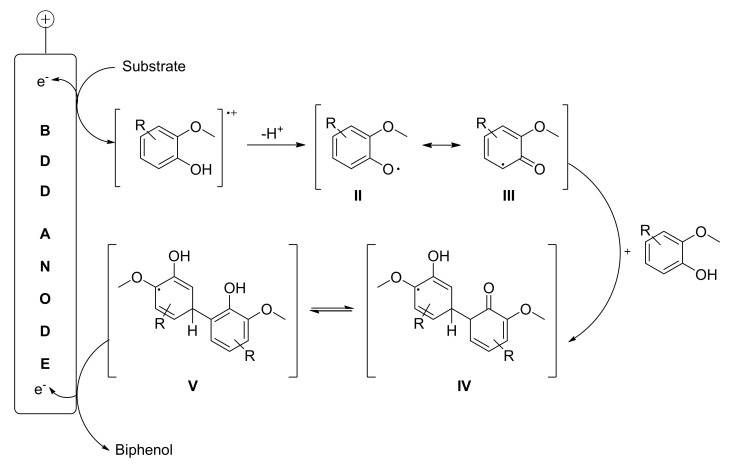
Proposed mechanism for guaiacol anodic coupling.

This reaction represented a significant breakthrough because it gave access to biaryl without the assistance of functional groups or the use of transition metals [26], resulting in a more atom-economy efficient and waste-free synthesis. The steric hindrance of the R group plays a fundamental role in dictating the final product of the reaction. If R is a bulky group, the formation of product **b** is favoured due to the less steric congestion in the final product. A mechanism was also proposed, and it is depicted in Scheme 3.

The radical cation, derived from the oxidation of the substrate by the BDD anode, is characterised by an enhanced acidity; therefore, a spontaneous removal of a proton can occur, obtaining the phenoxyl radical, **II**/**III**. The intermediate is trapped by another molecule of starting material to generate **IV** in tautomeric equilibrium with **V** that undergoes single electron transfer to afford the desired biphenol. When a bulky group is present, the phenoxyl radical is attacked by the guaiacol in the less hindered position, causing the formation of the non-symmetrical compound.

**Scheme 4:**
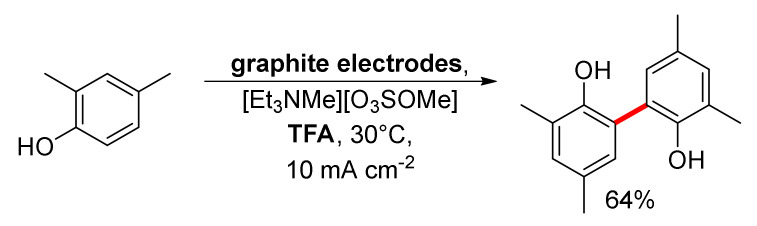
Updated conditions for the electrochemical synthesis of phenols.

Despite the good results obtained with the BDD/HFIP combination, Waldvogel’s group searched for a cheaper and green alternative to fluorinated alcohol and the boron-doped diamond [27]. Regarding the electrode, a suitable alternative was found to be graphite, known to be a trustworthy material for this kind of transformation [28,29]. However, when combined with HFIP, the reaction gave the desired product good yields (50–60%), plus a significant amount of polymeric by-products. Another media was needed, and after an intense optimization study, TFA was found to be the best candidate (Scheme 4).

With the updated conditions, the reaction selectively gave the desired product in good yield; moreover, the starting material was easily recovered by short-path distillation. An improvement of this reaction is discussed in a recent paper of 2019 by Selt et al. [30].

**Scheme 5:**
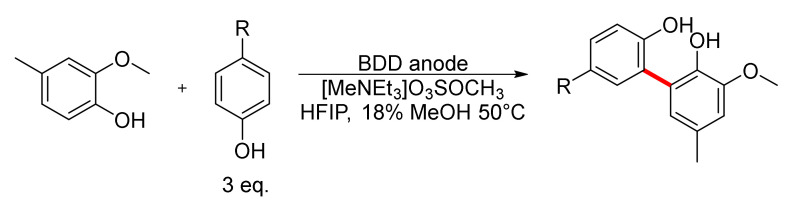
Non-symmetrical electrocoupling of phenols.

In 2014, Elsler et al. broadened the reaction scope (Scheme 5) and accomplished the synthesis of non-symmetrical biphenol [31].

To selectively obtain the non-symmetric product, the authors investigated the reaction conditions, and in particular, they used MeOH as an additive. In HFIP, methanol acts as a weak base interfering with the solvation and affecting the potential. Compared to other phenols, guaiacol has a minor oxidation potential, and with the addition of methanol, the potential difference is enhanced, lowering the guiacol one. Consequently, between the two reactions partners, the latter is be preferentially oxidised, and if phenol is present in large excess, the cross-coupling is the preferred final product. Besides, the low oxidation potential applied is not sufficient to oxidise the phenol, thus avoiding its homocoupling, even if present in excess. The reaction resulted suitable for a wide library of alkyl substituents as also some aryl moieties. As an additional value, in the paper, a greener synthetic route was developed; indeed, even if recyclable, fluorinated media still have a high environmental footprint. Preliminaries studies showed how formic acid could be a valuable alternative to HFIP [32]. Luckily, they were able to obtain the desired products, although in a lower yield, probably due to the inferior anodic stability of formic acid. Nevertheless, the low cost of the reactive compared to the fluorinated media make this result remarkable. 

In 2016, Wiebe et al. carried out studies on electrocoupling using as substrates protected phenols, different from the methyl ether derivatives typically used in the previous examples [33], making the methodology appealing for different synthetic strategies, especially in the field of total synthesis.

Between the different protecting groups, only alkyl or silyl derivatives were suitable. In the end, silyl ethers, in particular TIPS, resulted in being the best choice for their simple cleavage procedure compared to alkyl derivatives [34]. 

**Scheme 6:**
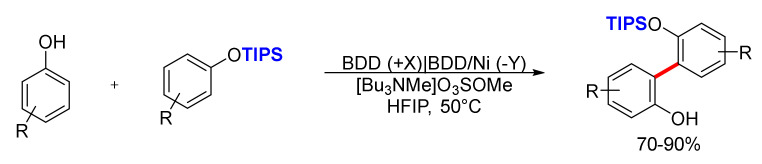
Non-symmetrical partially protected phenol electrocoupling.

**Figure 2:**
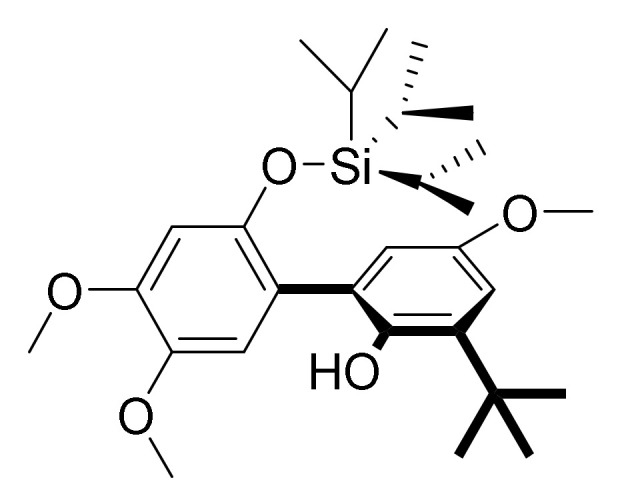
Molecular structure displaying the twist generated by TIPS.

The result showed in Scheme 6 highlights the robustness of the method. It is important to note that the TIPS also enhances the yields compared to the example with the unprotected phenols [31]. The authors explain these results as the combination of two different effects: the presence of a bulky group such as TIPS leads to a twist in the structure of the biphenol (Figure 2). 

This torsion is limited by an intramolecular hydrogen bonding between the OH and the silyl groups, to approximately 53°. In this conformation, the two π-systems are no longer conjugated. Therefore, the aryl moieties act as an EWG, protecting the final product from over oxidation. The second effect came from the solvation with HFIP. The two reaction partners are electron-rich systems that lead to strong solvation by the fluorinated media, which result in a decreased nucleophilicity penalising successive dehydrogenative couplings.

**Scheme 7:**
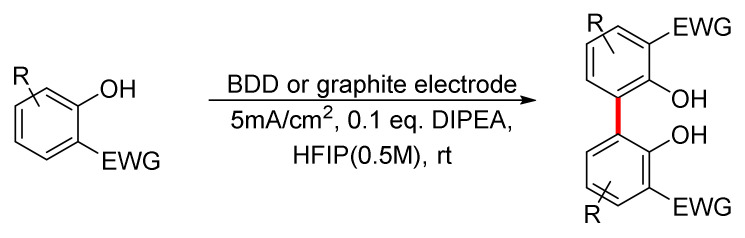
Optimised conditions for the coupling of electron-poor phenols.

Thus far, all the reported examples have been focused on electron-rich phenols because more prone to oxidation. However, electron-poor biphenols are also attractive targets but more difficult to synthetize [31,35]. In 2020, however, Röchl et al. developed a method able to overcome this limitation [36]. One of the principal changes was the use of DIPEA instead of the supporting electrolyte (Scheme 7). The base, other than facilitating the conductivity of the solution, can also lower the oxidation potential of the starting materials. Unfortunately, it promotes the formation of the HFIP-ether [37,38]; in order to suppress this phenomenon, a low current density must be applied, a catalytic amount of DIPEA is used, and the concentration of a high substrate is required.

**Scheme 8:**
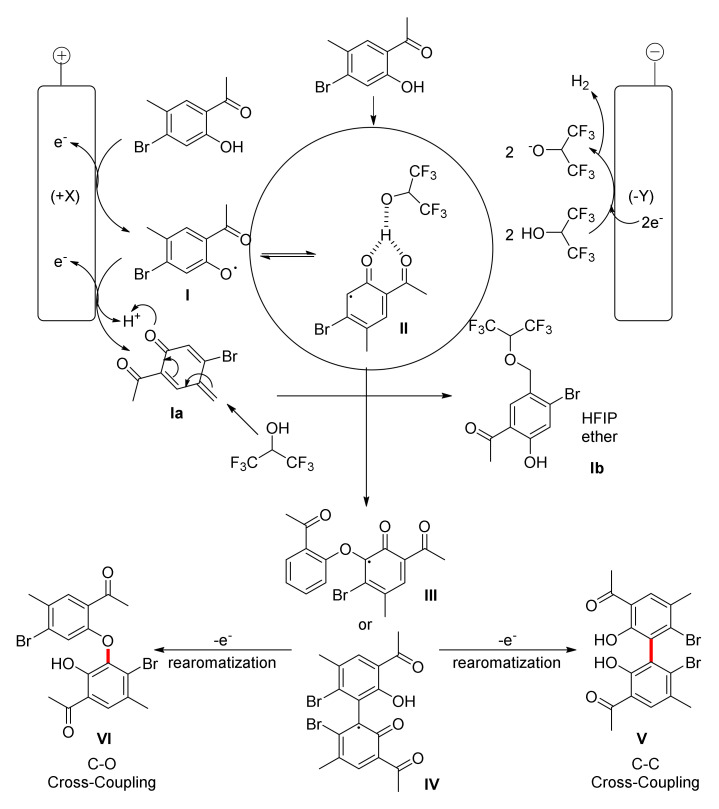
Proposed mechanism by Röckl et al.

With the exception of the aldehyde group, which easily gave over oxidation products, the method tolerates a wide variety of functional groups. Even if the yields are not excellent (30–64% range), the starting material can be recovered and recycled. Of note, if pyridine is used instead of DIPEA, the preferential product would be the C–O coupling. The mechanism proposed by the authors is presented in Scheme 8.

After the SET from the substrate to the anode, the radical **I** can tautomerise to the solvated intermediate **II** or undergo another single electron transfer, which will lead to the HFIP-ether **Ib**. Thanks to the high starting material concentration, the **Ib** synthetic route is discarded. Nucleophilic attack from another molecule of phenol can lend to the C–C or C–O cross-coupling product. At this point, loss of an electron and consequent rearomatisation gave the desired compound.

## 3. Naphthols Electrocoupling

**Scheme 9:**
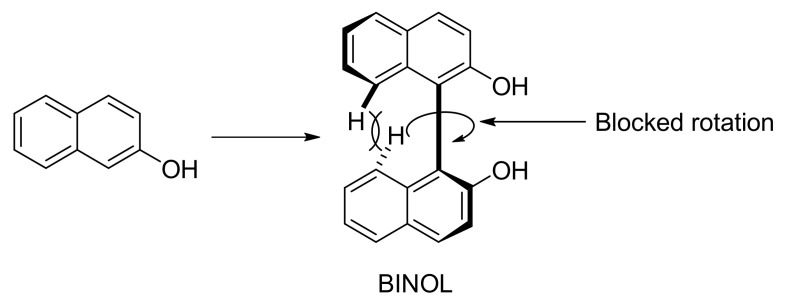
BINOL atropoisomerism.

BINOL and derivatives (Scheme 9) due to the possibility to be widely functionalised, and the intrinsic atropoisomerism had a big impact in the world of chiral ligands used in asymmetric catalysis, giving birth to different famous catalysts [39,40,41].

**Scheme 10:**
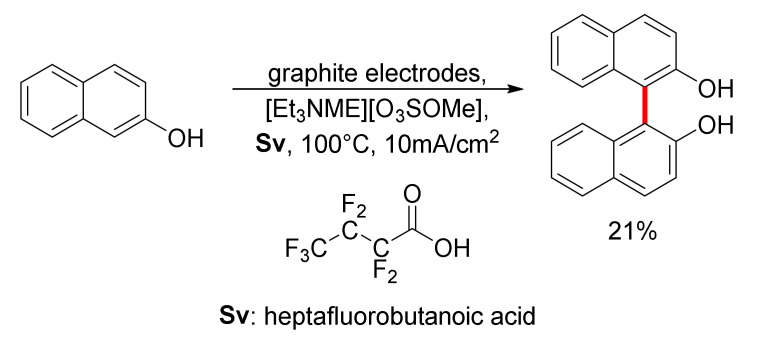
2-naphthol homocoupling conditions.

Nevertheless, for the synthesis of this very popular chiral scaffold, only organometallic or organocatalysed methods are known [41,42,43,44,45]. Electrochemistry represents a powerful and generally greener synthetic strategy; unfortunately, to the best of our knowledge, Waldvogel and co-workers, in a paper in 2011, presented in the previous section [23], reported only one example of 2-naphthol homo-electrocoupling (Scheme 10). Here, for the naphthol electrocoupling, TFA and HFIP were no more suitable media due to the insolubility of the substrates and the necessity to raise the temperature of the reaction. Heptafluorobutanoic acid resulted in being the best choice.

The desired product was obtained in low yields; indeed, compared to phenols, naphthols are more labile to this kind of conditions, which generate different by-products.

## 4. Aniline Electrocoupling

Similar to phenols, anilines are one of the most crucial building blocks in chemistry. They are found in natural products, materials, and many other fields [46,47]. It was only a matter of time before electrochemistry started to investigate this class of compounds. Biaryl derived from aminobenzenes find applications in different fields as auxiliary ligands [48] or contrast agents [49]. However, their synthesis remains quite challenging for modern chemists. One of the principal reasons is the tendency to form different by-products, including “aniline black” [50] derived from the oxidative polymerisation of the aniline. The principal synthetic methods use organometallic catalysis [51,52].

**Figure 3:**
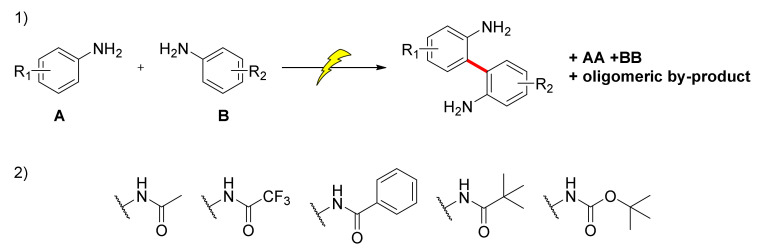
(**1**) Direct oxidation of aniline. (**2**) Amine protecting group used by Waldvogel and co-workers.

In 2017, Waldwogel and co-workers were able to synthetize 2,2′-diamino biaryls with a metal-free protocol (Figure 3) [53]. However, some precautions are needed compared to the reaction with phenols; indeed, the low oxidation potential typical for this substrate promotes overoxidation, with consequent polymerisation [54]. In a recent publication, N-metahanesulfonyl protected anilines were successfully employed in the coupling with different arenes. Of note, the use of other protecting groups, such as acetate or trifluoroacetate, Boc, and triflic groups, did not afford any of the desired coupling products [55].

**Scheme 11:**
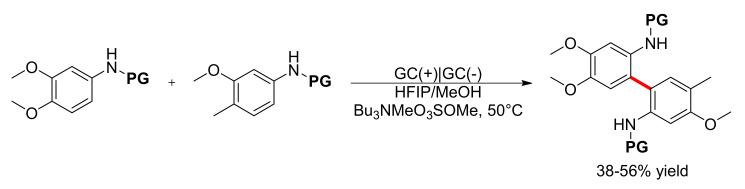
Anodic oxidation of N-protected anilines.

CV studies of the *N*-protected anilines show an increase in the oxidation potential; for example, 3,4-dimethoxyaniline has an oxidation potential of 832 mV in pure HFIP, while the potential of its *N*-acetyl derivative is raised to 1.39 V (vs. Ag/AgCl in saturated LiCl/EtOH), making the system less prone to over oxidation. Another tuneable parameter is the solvation. Anilines are both good acceptors and donors of H-bonds. Considering the impressive solvation of HFIP, a strong interaction is formed between the solvent and the reactant, with a dramatic lowering of the oxidation potential. For this reason, the addition of MeOH is required to disrupt the strong H-bond interaction and modulate the potential. In this reaction, a considerable role is also played by the supporting electrolyte (Scheme 11).

The reaction gave moderate yields, but it is possible to recover the starting material during the purification. Moreover, no deprotection during the reaction was found to occur, regardless of the protective group that was used.

**Scheme 12:**
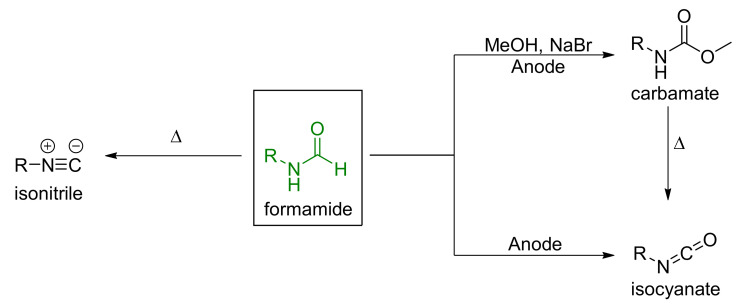
Synthetic importance of formamides.

It is undoubtedly that a protecting group is necessary, so the Waldvogel group makes an intensive study on *N*-formyl derivatives (Scheme 12) [56], considering their synthetic interest [57,58] and being the formyl group an atom-economic protecting group [59].

The methodology worked well also for this specific protecting group without observing degradation or deprotection. In addition, halogen-containing formanilides were used, and no side reactions or dehalogenation were observed.

**Scheme 13:**
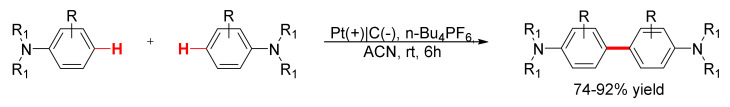
Benzidine anodic coupling.

Thus far, the reported coupling reactions were selective for the ortho–ortho C–C coupling. Thanks to the work of Gui and co-workers, it is now possible to have access to benzidine by electrochemical methods (Scheme 13) [60]. Moreover, these compounds, also known as 4,4′-diaminobiaryl, are found in various pharmaceutical products [61,62] and as building blocks for the synthesis of heterocycles [1,63].

**Figure 4:**
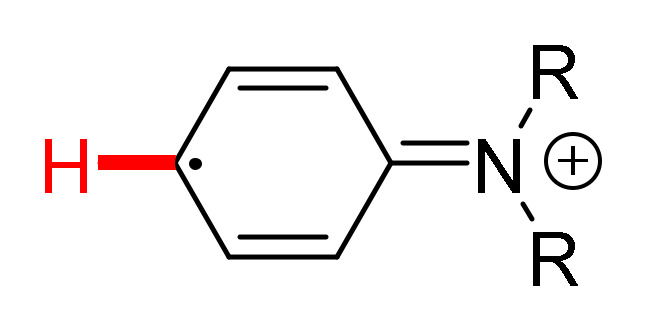
*Para-radical* cation intermediate.

Aliphatic and aromatic *N*-protected anilines were also used with EWG and EDG groups as halogens. In addition, bulky protecting groups did not influence the overall yield. The authors stated that no *ortho* or *meta* coupling products were observed due to the high stability of the *para*-radical cation intermediate (Figure 4).

## 5. Unsymmetrical Biaryl Electrosynthesis

**Figure 5:**
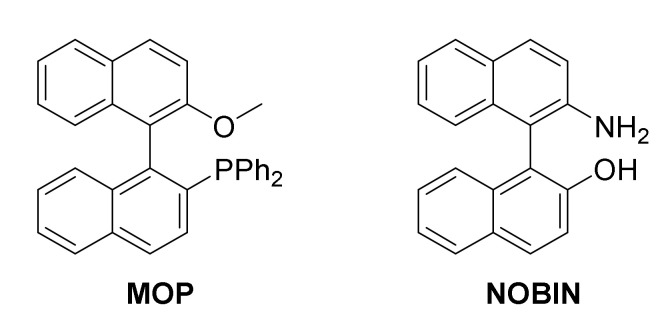
Chemical structure of MOP and NOBIN.

In the previous sections, homocoupling of different classes of compounds was discussed; however, unsymmetrical biaryls are also important and useful compounds (Figure 5). In the early 1990s, Hayashi [64], with MOP and Kocovsky [65] with NOBIN, gave birth to a new family of biaryl compounds that are applied in catalysis [66,67] and also in medicinal chemistry [68,69]. 

**Scheme 14:**
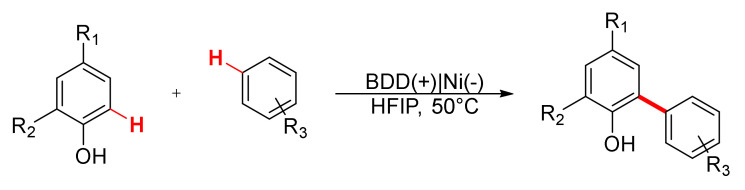
Phenol–Arene Anodic Cross-Coupling.

Anodic treatment of arenes normally led to the starting material’s homocoupling, and only a few examples gave the desired cross-coupling product [70]. Once again, the situation changed when the BDD electrode and fluorinated solvent were introduced (Scheme 14). In 2010, Waldvogel and co-workers devised a procedure with high selectivity toward cross-coupling [71].

Compared to previous works, the best results and selectivity were obtained with the conditions shown in Scheme 14, with a lower current density, only 2.8 mA cm^−2^. The authors highlight the importance of the BDD electrode; indeed, when carbon-based electrodes were tested, only homo-dehydro dimers were found. A few years later, Waldvogel and co-workers improved their methodology with the introduction of additives as MeOH and H_2_O [72], whose effects were explained in the previous section.

With the idea to create a greener and cheaper protocol, Atobe and co-workers [73] looked for alternatives to the use of HFIP due to its high cost and non-negligible environmental footprint [74]. In order to mimic the effect of the perfluorinated alcohol, a solvent with a high acceptor number [75] is needed in order to stabilise the radical cation intermediate. Solvents with a high acceptor number (A.N.) are less prone to make a nucleophilic attack on the intermediate dystonic ion, extending its lifetime [76]. Methanol as an additive increased the selectivity between homo/cross-coupling (Table 1).

**Scheme 15:**
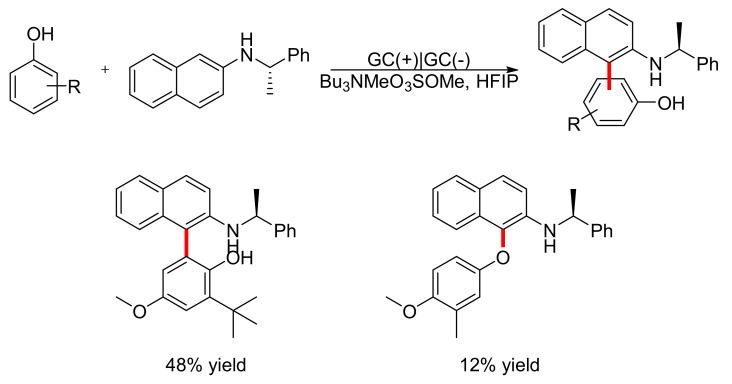
2-Naphthylamine-phenol cross-coupling.

In order to complete the portfolio of unsymmetrical electrocoupling reactions, several research groups introduced a different coupling partner other than arenes (Scheme 15). Once again, Waldvogel and co-workers, pioneer group in electrochemistry, opted to use 2-naphtylamine [77]. This class of compounds is relatively unexplored in the field of electrocoupling. For the homocoupling, only a paper from 1979 is known [78].

Besides the desired product, obtained in moderate yields, it was also noticed, in some cases, the formation of the C–O cross-coupling product. In-deep investigations pointed out that the steric hindrance present on the ortho position of phenol is responsible for the final product structure. High steric hindrance leads to the C–C coupling product, and low steric hindrance favours the C–O connected compound. A drop in the yields was observed, probably due to the formation of a benzylic radical that is prone to side reactions, for phenols bearing methyl or ethyl moieties in *para* position.

**Scheme 16:**
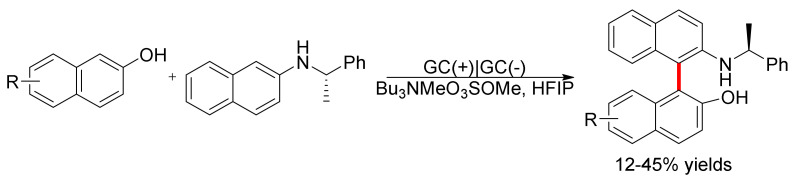
2-naphtalymine 2-naphthol cross-coupling.

To complete their investigation, they also decided to analyse the cross-coupling between naphthylamines and naphthol (Scheme 16).

The setup was slightly different due to the minor electrochemical robustness of naphthol compared to phenol, being more prone to over-oxidation and side reaction. It was necessary to lower the current density and the temperature. On the other hand, an excess of alcohol was not necessary to ensure the cross-coupling, and, in all the cases, no C–O coupling was observed. 

With the idea to demonstrate the potentiality of this reaction and to broaden the scope, Waldvogel’s group investigated the functionalisation of benzofurans [79]. Their interest in this particular class of substances was born from the ubiquity of benzofurans in natural products and bioactive molecules [80,81,82]. 

**Scheme 17:**
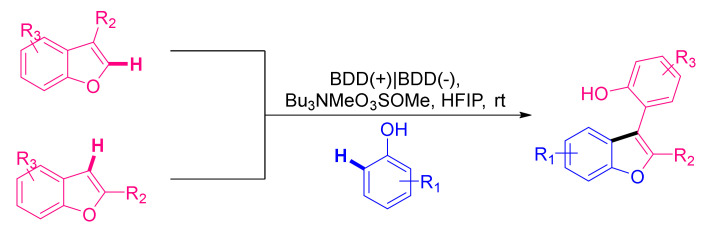
Benzofuran phenol cross-coupling.

**Scheme 18:**
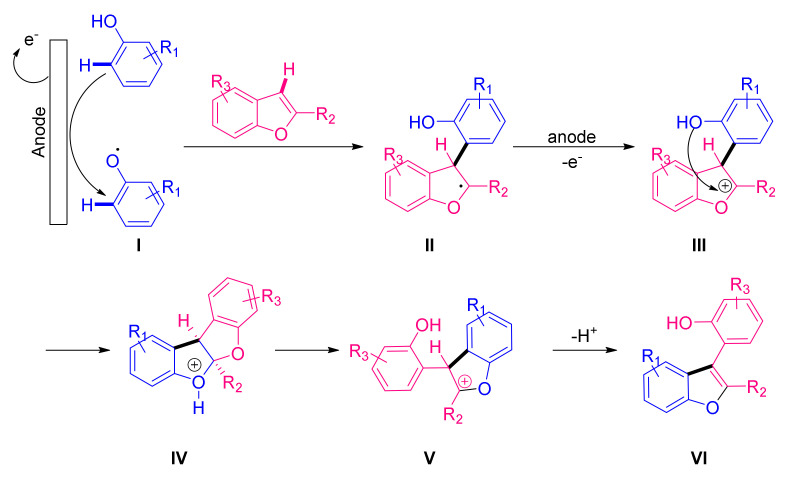
Proposed mechanism for the cross-coupling between phenol and **2**-substituted benzofuran.

Although the cross-coupling succeeded, the authors were quite surprised by the furan metathesis usually observed in all the reactions (Scheme 17). The data collected from the isolation of some intermediates suggest two different mechanisms, one starting from the 2-substituted benzofuran and one for 3-substituted one. The mechanism for the cross-coupling between phenol and 2-substituted benzofuran is reported in Scheme 18.

SET between the phenol, lowest oxidation potential, and the anode generate the phenoxyl radical that undergoes nucleophilic attack by the benzofuran. The neutral radical **II** is subject to another SET generating the carbocation in position 2 of the benzofuran moiety. Nucleophilic attack from the hydroxyl group affords the protonated dihydrobenzofuro [2,3-*b*]benzofuran(**IV**). Rearrangement to the most stable carbocation (**V**) by ring-opening and consequent deprotonation led to the final product.

**Scheme 19:**
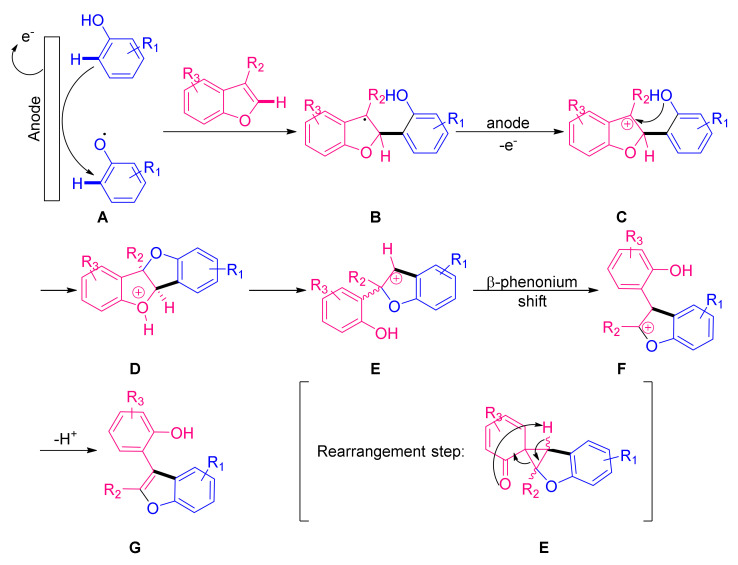
Proposed mechanism for the cross-coupling between phenol and **3**-substituted benzofuran.

Regarding the 3-substituted benzofuran, the first steps are the same (Scheme 19); as the cationic intermediate **C** is formed, it undergoes an intramolecular attack at position 3 of the furan ring. The key intermediate **D** is the dihydrobenzofuro [3,2-*b*]benzofuran and, as with **IV** ring-opening, evolves to the most stable carbocation. However, at this point, a 1,2-phenyl shift occurred due to the steric hindrance in position 2. Deprotonation of **F** and consequent rearomatisation give the desired compound. Waldvogel and co-workers noticed how the driving force of the mechanism is the greater stability of the carbocation **V/E** compared to **IV/D**.

**Figure 6:**
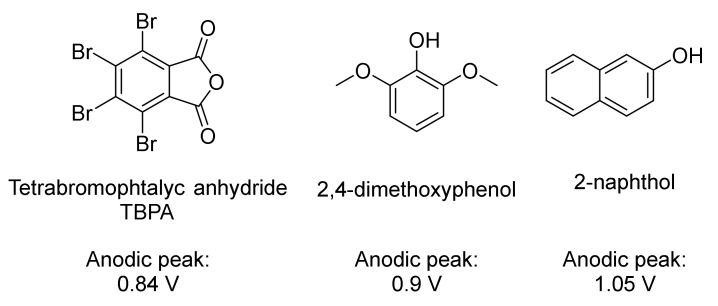
Oxidation potential of TBPA, 2,6-dimethylphenol, 2-naphthol. CV conditions: 0.1 mol/L LiClO_4_/CH_3_CN; Pt disk w. e.; Ag wire c. e.; Ag/AgNO_3_ 0.1 mol/L in CH_3_CN ref. e.

It is worth mentioning the work published by the group of Sun in 2019 [83]. They investigated the cross-coupling between phenols and 2-naphthol in the presence of a redox mediator: tetrabromophtalic anhydride, TBPA (Figure 6). Thanks to CV studies, they disclosed the ability of TBPA to act as an intermediary.

Indeed it has the lowest oxidation potential compared to 2,6-dimethoxyphenol and 2-naphthol. The slight potential difference between TBPA and the phenol suggests that the mediator’s radical cation form will have a SET preferentially with the phenol generating the corresponding radical. The mediator strategy gave excellent results with an increased yield, up to 83%, and a drastic enhancement of the selectivity towards the cross-coupling product. Furthermore, this methodology allowed the bis-arylation, in positions 1 and 4, when naphthol-2,3-diol is used.

**Figure 7:**
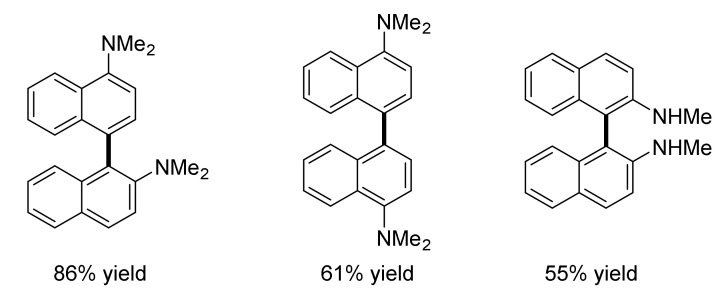
Naphthylamines cross/homo-coupling.

Other than aromatic alcohols, also the nitrogen-containing counterpart was intensively studied. In 2020, the group of He developed a protocol for the cross-coupling between aniline and 2-naphthylamine [84]. The method is compatible with a wide variety of functional groups. It was possible to obtain also the product of cross/homo-coupling between naphthylamines (Figure 7), which was only partially achieved electrochemically in two papers of 1980 [78,85].

## 6. Miscellaneous

In this final section, we focus our attention on a few interesting papers. In 2019, the group Chen published their studies on electrochemical regioselective C(sp^2^)-H azolation of phenols [86]. Indeed, N-arylated heterocycles are well-known in organic chemistry for their presence in natural products [87,88] and as functional material [89]. Although it is easy to oxidise both phenols and azoles electrochemically, their electro-combination was not achieved at the time [90,91]. Instead, this new reaction allows synthesising the desired product in high yields and with excellent regioselectivities.

**Scheme 20:**
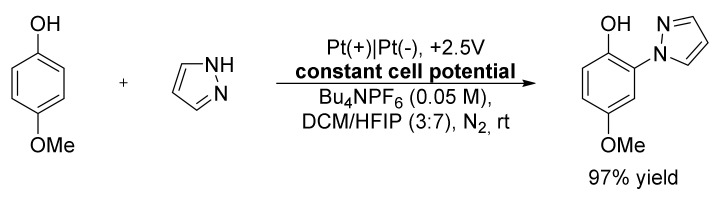
Electrochemical N-arylation of Azole.

The electrochemical conditions deserve a special comment. As depicted in Scheme 20, the reaction was conducted in potentiostat conditions instead of galvanostatic. Electrolysis with constant potential lends to the maximum selectivity because the potential of the reaction is set equal to that of the starting material but is generally slower and with a more complicated setup (working electrode, counter electrode, reference electrode) [73]. The reaction also worked with anilines, and excellent yields were obtained with a high tolerance of different functional groups. Further control experiments were carried out to gain some insights into the mechanism. From them, they established that: (a) Phenol does not form the corresponding dystonic cation. Instead, it is the pyrazole to undergo oxidation; (b) In the case of aniline, experiments suggest the formation of N-centered radical; (c) Michael’s addition pathway must be excluded.

**Scheme 21:**
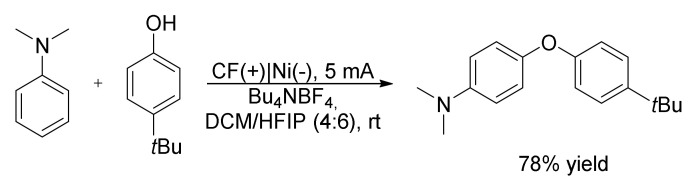
Diaryl ether electrosynthesis.

The other mention is dedicated to a recent paper from the group of Vercammen [92]. Unlike the other examples reported in the previous sections, in which the C–C coupling was the objective, the authors developed a methodology for the C–O coupling between phenol and aniline to afford diaryl ethers, important compounds as bioactive molecules [93,94,95]. In order to enhance the yield of the desired product and based on the results obtained by Waldvogel in some isolated cases [36,77], they opted for the following substrates: phenols with low steric hindrance, aniline with *para* position activated, and with a low oxidation potential (Scheme 21).

**Figure 8:**
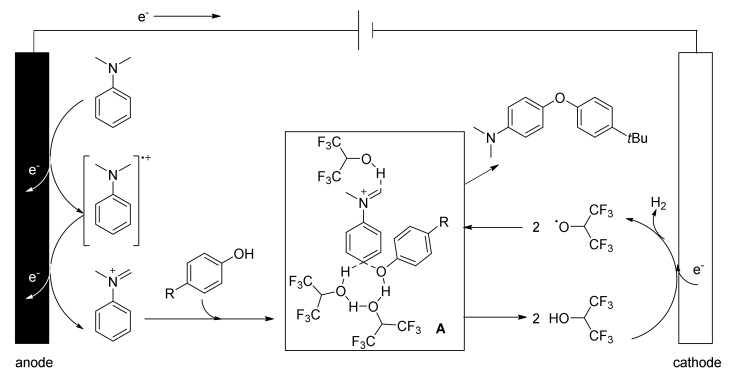
Proposed mechanism for C(sp^2^)-O coupling.

When only DCM or HFIP were used, a drastic yield drop is observed, as with MeOH and CAN; the reaction also resulted quite sensible to the cathode material. Indeed, when a Pd electrode was used, a decrease in yield was observed. This behaviour can be ascribed to a reduction step, fundamental for the mechanism, highly dependent on the surface properties of the electrode. Homocoupling, one of the principal parasite reactions in electrochemical reactions, was observed in rare cases and only in traces. CV and EPR analysis of the reaction crude confirmed that the first species to be oxidised is the aniline. The following mechanism was then proposed (Figure 8).

Anodic oxidation of the tertiary aniline generates the corresponding cation that is attacked by the phenol. Besides decreasing the nucleophilicity and the possibility of side-reactions, HFIP acts as a mediator between the cathode and the solvated intermediate **A**, facilitating the hydrogen transfer necessary to obtain the final product. High tolerance to a wide variety of functional groups was observed.

## 7. Conclusions

In the last decade, electrochemistry has become a valuable method for the synthesis of biaryl, thanks to the introduction of BDD as a new electrode combined with the versatility of the fluorinated media, especially HFIP. The efforts of different groups resulted in the development of a variety of new reactions applicable to a vast library of biaryl substrates. In addition, the reagent role is taken by electrons instead of oxidising or reducing agents, which makes the reaction greener thanks to the decreased production of waste.

In the case of aryl coupling, efficient methodologies for the enantioselective synthesis of not-racemic biaryl are clearly underdeveloped. In 1994, the electrocatalytic oxidative coupling of naphthol on a TEMPO-modified felt electrode in the presence of sparteine was described [96]. However, since then, no further examples have been reported, and new contributions in the area are expected and are largely desirable.

As regards the future, we also foresee a major entanglement between flow chemistry and electrochemistry. The new asset will further increase the attractiveness of electrosynthesis [97,98,99]. The chemistry is also evolving toward a greener approach; therefore, new eco-friendly protocols could be envisioned, such as the one where Deep Eutectic Solvents were used as green media [100,101].

## Figures and Tables

**Table 1:** Acceptor Number of some of the solvents considered in Atobe’s work.

Solvent	Acceptor Number
HFIP	88
AcOH	52.9
HCOOH	83.6
TFA	105

## Data Availability

Not applicable.
